# Single-Cell Transcriptome Analysis Decipher New Potential Regulation Mechanism of ACE2 and NPs Signaling Among Heart Failure Patients Infected With SARS-CoV-2

**DOI:** 10.3389/fcvm.2021.628885

**Published:** 2021-02-23

**Authors:** Mengqiu Ma, Yanhua Xu, Yang Su, Sang-Bing Ong, Xingdong Hu, Min Chai, Maojun Zhao, Hong Li, Xiaojuan Fan, Yingjie Chen, Dachun Xu, Xiaojiang Xu

**Affiliations:** ^1^Department of Cardiology, Shanghai Tenth People's Hospital, Tongji University School of Medicine, Shanghai, China; ^2^Centre for Cardiovascular Genomics and Medicine (CCGM), Lui Che Woo Institute of Innovative Medicine, Chinese University of Hong Kong (CUHK), Hong Kong, China; ^3^Hong Kong Hub of Paediatric Excellence (HK HOPE), Hong Kong Children's Hospital (HKCH), Hong Kong, China; ^4^Department of Medicine and Therapeutics, Faculty of Medicine, Chinese University of Hong Kong (CUHK), Hong Kong, China; ^5^Institute for Translational Medicine, Xiamen Cardiovascular Hospital, Xiamen University, Xiamen, China; ^6^Kunming Institute of Zoology–The Chinese University of Hong Kong (KIZ-CUHK) Joint Laboratory of Bioresources and Molecular Research of Common Diseases, Kunming Institute of Zoology, Chinese Academy of Sciences, Kunming, China; ^7^Department of Critical Care Medicine, The Third people's Hospital of Guizhou Province, Guiyang, China; ^8^Department of Critical Care Medicine, Ezhou Central Hospital, Ezhou, China; ^9^Emergency Department, The First People's Hospital of Guiyang, Guiyang, China; ^10^Immunity, Inflammation & Disease Laboratory, The National Institute of Environmental Health Sciences, National Institutes of Health, Durham, NC, United States; ^11^Key Laboratory of Environment and Genes Related to Diseases, Department of Cardiology, First Affiliated Hospital of Xi'an Jiaotong University, Xi'an, China; ^12^Department of Physiology & Biophysics, University of Mississippi Medical Center, Jackson, MS, United States; ^13^Kelly Government Solutions, Rockville, MD, United States

**Keywords:** COVID-19, SARS-CoV-2, heart failure, angiotensin converting enzyme 2, single-cell RNA sequencing

## Abstract

**Aims:** COVID-19 patients with comorbidities such as hypertension or heart failure (HF) are associated with poor clinical outcomes. The cellular distribution of Angiotensin-converting enzyme 2 (ACE2), the critical enzyme for SARS-CoV-2 infection, in the human heart is unknown. We explore the underlying mechanism that leads to increased susceptibility to SARS-CoV-2 in patients with cardiovascular diseases and patients of cardiac dysfunction have increased risk of multi-organ injury compared with patients of normal cardiac function.

**Methods and Results:** We analyzed single-cell RNA sequencing (scRNA-seq) data in both normal and failing hearts. The results demonstrated that ACE2 is present in cardiomyocytes (CMs) and non-CMs, while the number of ACE2-postive (ACE2+) CMs and ACE2 gene expression in these CMs are significantly increased in the failing hearts. Interestingly, both brain natriuretic peptides (BNP) and atrial natriuretic peptide (ANP) are significantly up-regulated in the ACE2+ CMs, which is consistent with other studies that ACE2, ANP, and BNP increased in HF patients. We found that genes related to virus entry, virus replication and suppression of interferon-gamma signaling are all up-regulated in failing CMs, and the increase was significantly higher in ACE2+ CMs, suggesting that these CMs may be more vulnerable to virus infection. As the level of expression of both ACE2 and BNP in CMs were up-regulated, we further performed retrospective analysis of the plasma BNP levels and clinical outcomes of 91 COVID-19 patients from a single-center. Patients with higher plasma BNP were associated with significantly higher mortality and expression levels of inflammatory and infective markers.

**Conclusion:** In the failing heart, the upregulation of ACE2 and virus infection associated genes could potentially facilitate SARS-CoV-2 virus entry and replication in these vulnerable cardiomyocyte subsets. COVID-19 patients with higher plasma BNP levels had poorer clinical outcomes. These observations may allude to a potential regulatory association between ACE2 and BNP in mediating myocarditis associated with COVID-19.

## Introduction

Novel coronavirus disease 2019 (COVID-19) is an infectious disease caused by severe acute respiratory syndrome coronavirus 2 (SARS-CoV-2) (1). As of January 2021, more than 102 million cases of COVID-19 and more than 1 million deaths have been reported worldwide (2). In addition to the severe lung infection, the SARS-CoV-2 virus also causes myocarditis, cardiac dysfunction, and heart failure (HF) (1, 3–5). Conversely, COVID-19 patients with pre-existing conditions, such as hypertension and coronary heart disease, have worse clinical outcomes than those without these comorbidities (5–7). In this regard, these findings may point towards the presence of a vicious cycle between SARS-CoV-2 infection and cardiac dysfunction or HF (6).

Angiotensin-converting enzyme 2 (ACE2) is the critical enzyme degrading the pro-inflammatory angiotensin-II (Ang II) to the anti-inflammatory Ang 1–7 (8, 9). Unfortunately, ACE2 also facilitates SARS-CoV-2 entry into host cells by binding its surface “spike” protein. ACE2 is highly expressed in the nose, kidney, intestine, colon, brain, endothelium, testis, and heart (10–15). A recent study from Zou et al. reported that ~7% cardiomyocytes (CMs) express ACE2 in normal human cardiac tissues (12), suggesting that some CMs can be directly infected by SARS-CoV-2. However, ACE2 gene expression in different cardiomyocyte subsets, as well as its dynamic changes in failing human hearts at the single cell level, are totally unknown.

Since ACE2 plays an important role in SARS-CoV-2 infection and cardiac function, it is critically important to understand its distribution and the biological changes associated with its altered expression in normal and failing hearts. Therefore, we investigated the ACE2 gene expression profiles by analyzing the single-cell RNA sequencing (scRNA-seq) dataset derived from both normal and failing human hearts (16). We found that ACE2 was expressed in CMs, vascular endothelial cells, fibroblasts, smooth muscle cells and immune cells in normal hearts, and its expression was further increased in several cell subsets in the failing hearts. Importantly, we found that brain natriuretic peptide (BNP) and atrial natriuretic peptide (ANP) (NPs) transcripts are co-upregulated in ACE2-postive (ACE2+) CMs. NPs and ACE2 may form a feedback loop associated with the rein-angiotensin-aldosterone-system (RAAS)/Ang II signaling pathway. Furthermore, ACE2 expression was also associated with the dynamic changes of a group of genes related to viral infection and acquired immunity. Since there is a positive correlation between the expressions of BNP and ACE2, we further analyzed the clinical outcome, inflammation markers, and blood BNP levels in COVID-19 patients retrospectively. Together, these findings provide important insights to advance our understanding of the interplay between ACE2, viral infection and inflammation, as well as cardiac injury and failure.

## Materials and Methods

### Clinical Study

#### Design, Participants, and Data Collection

This is a retrospective, single-center study including 91 patients with laboratory-confirmed COVID-19 admitted to Ezhou Central Hospital, Ezhou, China from January 25, 2020 and March 30, 2020. All procedures were followed in accordance with institutional guidelines. PCR-Fluorescence probing based kit (Novel Coronavirus (2019-nCoV) Nucleic Acid Diagnostic Kit, Sansure Biotech, China) was used to extract nucleic acids from clinical samples and to detect the ORF1ab gene (nCovORF1ab) and the N gene (nCoV-NP) according to the manufacturer's instructions. SARS-CoV-2 infection was laboratory-confirmed if the nCovORF1ab and nCoV-NP tests were both positive results. The study protocol was approved by the ethics committee of Shanghai Tenth People's Hospital, Tongji University School of Medicine (Shanghai, China) (Approval No. SHSY-IEC-4.1/20-63/02) and the ethics committee of Ezhou Central Hospital (Hubei, China) (Approval No. L2020-Y-013). Patient informed consent was waived by each ethics committee due to the COVID-19 pandemic.

COVID-19 was diagnosed by meeting at least one of these two criteria: (i) chest computerized tomography (CT) manifestations of viral pneumonia; and/or (ii) reverse transcription-polymerase chain reaction (RT-PCR) according to the New Coronavirus Pneumonia Prevention and Control Program (5th edition) published by the National Health Commission of China (New Coronavirus Pneumonia Prevention and Control Program, 2020) and WHO interim guidance (17). We used the following inclusion and exclusion criteria to determine the study cohort. The inclusion criteria were confirmed COVID19, valid BNP level and age above 18 years. The exclusion criteria were incomplete medical records, pregnancy, preexisting valvular heart disease, congenital heart disease, acute myocardial infarction, acute pulmonary embolism, acute stroke, HIV infection or conformed other virus infection, and end-stage cancer.

The demographic characteristics and clinical data (comorbidities, laboratory findings, and outcomes) for participants during hospitalization were collected from electronic medical records. Cardiac biomarkers measured on admission were collected, including Troponin I (TNI), creatine kinase-MB (CK-MB), and BNP. All data were independently reviewed and entered into the computer database by three analysts. Since the echocardiography data were unavailable for most patients, patients were categorized according to the BNP level. Acute HF was defined as a blood BNP level ≥100 pg/ml. The clinical outcomes (i.e., discharges and mortality) were monitored up to 30 days.

#### Statistical Analysis

Descriptive statistics were obtained for all study variables. Continuous data were expressed as mean [standard deviation (SD)] or median [interquartile (IQR)] values. Categorical data were expressed as proportions. All continuous variables were compared using the *t*-test or the Mann–Whitney *U*-test if appropriate. In contrast, categorical variables were analyzed for the study outcome by Fisher exact test or χ^2^ test. The Pearson correlation coefficient and Spearman rank correlation coefficient were used for linear correlation analysis. Survival analysis between patients with BNP<100 pg/mL and ≥100 pg/mL was conducted by the Kaplan-Meier estimate with *p*-value generated by the log-rank test. Data were analyzed using SPSS version 25.0 (IBM Corp) or Graphpad Prism 8.0.1 (GraphPad Software, San Diego, CA). For all the statistical analyses, 2-sided *p* < 0.05 was considered significant.

### scRNA-Seq Analysis

#### Data Sources

Adult human heart scRNA-seq datasets were obtained from Gene Expression Omnibus (GEO) under accession codes GSE109816 and GSE121893. Briefly, samples from twelve healthy donors and samples from six patients with HF were collected at the time of heart transplantation. The range of donor ages was 21–52 year, with a median age of 45.5 years.

#### Sequencing Data Processing

The processed read count matrix was retrieved from existing sources based on previously published data as specified explicitly in the reference. Briefly, Raw reads were processed using the Perl pipeline script supplied by Takara.

#### Single-Cell Clustering and Identified Cell Types

The processed read count matrix was imported into R (Version 3.6.2) and converted to a Seurat object using the Seurat R package (Version 3.1.2). Cells that had over 75% UMIs derived from the mitochondrial genome were discarded. For the remaining cells, gene expression matrices were normalized to total cellular read count using the negative binomial regression method implemented in Seurat *SCTransform* function. Cell-cycle scores were calculated using Seurat *CellCycleScoring* function. The Seurat *RunPCA* functions were performed to calculate principal components (PCs). We further corrected the batch effect using Harmony because batch effects among the human heart samples were observed. The *RunUMAP* function with default setting was applied to visualize the first 35 Harmony aligned coordinates. The *FindClusters* function with resolution=0.2 parameter was carried out to cluster cells into different groups. Canonical marker genes were applied to annotate cell clusters into known biological cell types. Monocle 3 as used to perform trajectory and pseudotime analysis.

#### Identification of Differential Expression Genes (DEGs)

To identify DEG between two groups, we applied the Seurat *FindMarkers* function with the default parameter of method “MAST” and cells ID from each defined group (e.g., ACE2+ cells versus ACE2 negative (ACE2-) cells in CM1) as input.

#### Gene Function Analysis

GSEA (Version 4.03) was used to perform gene ontology (GO) term and pathway enrichment analysis with the Molecular Signatures Database (MSigDB, C2 and C5, Version 7.01).

## Results

### Integrated Analysis of Normal and HF Conditions at Single-Cell Resolution

**Figure 1:**
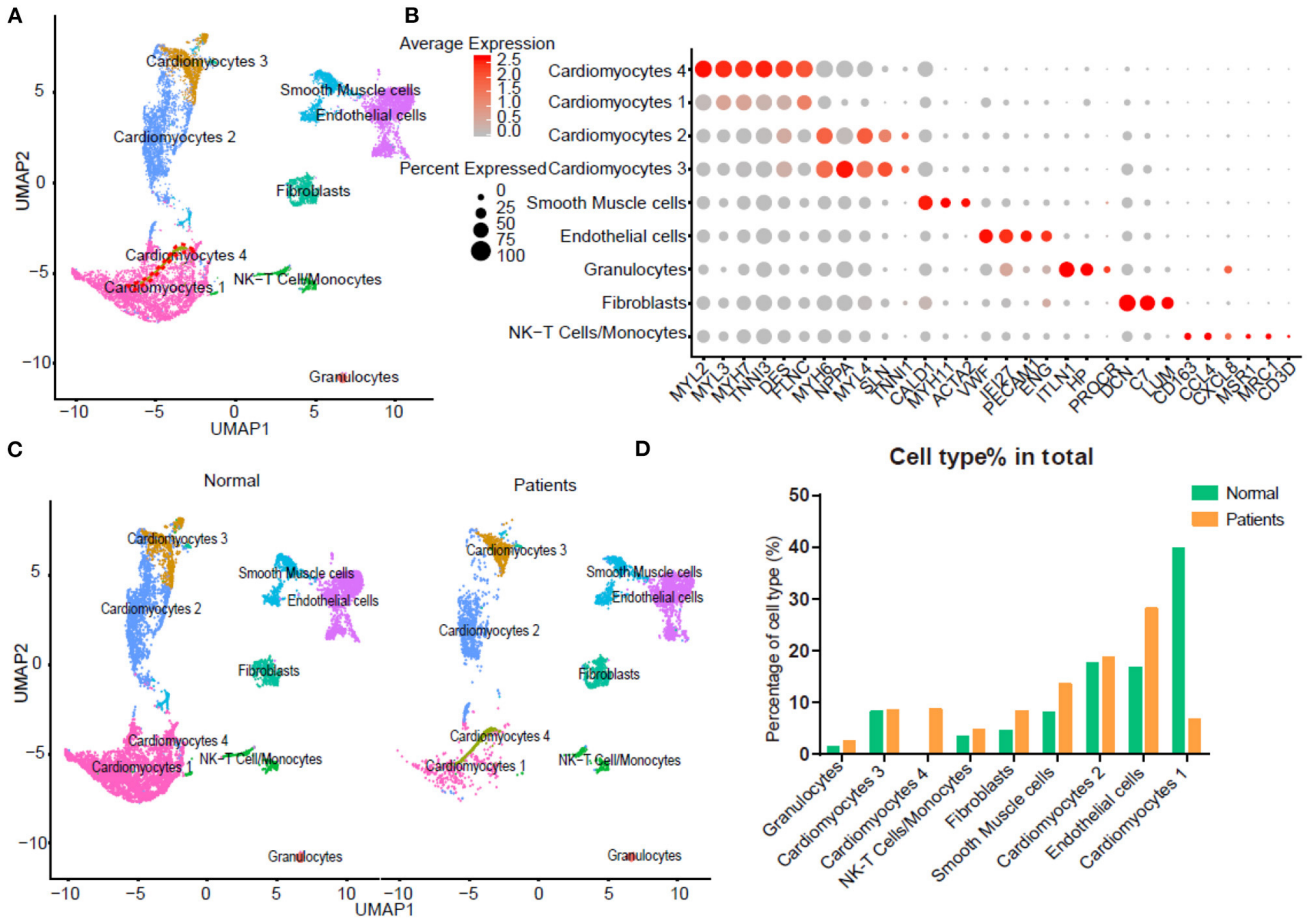
Integrated analysis of normal and heart failure (HF) conditions at single-cell resolution. **(A)** Uniform manifold approximation and projection (UAMP) clustering of 14,698 cells isolated from normal and heart failure patients. Each dot represents a single cell. Cell type was annotated by the expression of known marker genes. **(B)** Dot plotting showing gene signature among different clusters, the shadings denotes average expression levels and the sizes of dots denote fractional expression. **(C)** Split views show the 9 subsets in normal and patient group. **(D)** The percentage of cell number for different cell types in normal and patient group.

To detect the discrepancy between normal and HF patients, we utilized the scRNA-seq data by Wang et al. (16). Briefly, twelve control samples were collected from healthy donor hearts (hereinafter called normals). Samples from six HF patients were collected at the time of heart transplantation. Thirteen thousand nine hundred eighty-six out of 15,215 cells passed standard quality control and were retained for subsequent analyses. After UMAP and clustering analysis the entire cell population were grouped into nine subsets (Figure 1A). Dot plot showed the expression of known markers for nine clusters, which included: (1) endothelial cells (PECAM1 and VWF); (2) fibroblasts (LUM and DCN); (3) smooth muscle cells (MYH11); (4) NK-T/ monocytes (CD3G and CD163); (5) granulocytes (HP and ITLN1); (6) CM2 and 3 subsets (MYH6 and NPPA); (7) CM1 and 4 subsets (MYH7 and MYL2) (Figure 1B). UMAP for individual sample exhibited the differential distribution of subsets between normal and HF patients (Figure 1C, Supplementary Table 1). As shown in Figure 1C, all nine subsets were detected in both normal and patient groups. However, the percentage of CM1 was dramatically decreased (*p* < 0.0001), while the percentage of CM4 was significantly increased (*p* < 0.0001) in HF samples. In addition, the percentages of CM2 (*p* > 0.05) and CM3 (*p* > 0.05) were not changed significantly. The percentages of vascular endothelial cells (*p* < 0.0001) and fibroblasts (*p* < 0.0001) were also significantly increased in the failing hearts (Figure 1D).

For each cluster, we calculated the cluster-specific genes (marker genes). Left ventricle (LV) marker genes MYL2 and MYL3 were highly expressed in CM1 and CM4; these subsets were thus termed ventricular cardiomyocytes. Since the left atrial (LA) marker genes MYH6 and MYH7 were highly expressed in CM2 and CM3 subsets, they were termed atrial CMs (18).

### Both CMs and Non-CMs (NCMs) Show Different Characteristics Between Normal and HF Patients

We compared gene expression of atrial CMs (CM2&3) and NCMs between normal and patients. We observed that GO term viral gene expression was up-regulated in all atrial CMs and NCMs in HF (Supplementary Figures 1A–F). These findings suggested that some CMs and NCMs in the heart may be liable to SARS-CoV-2 infection. In addition, GO results showed that genes related to the mitochondrial respiratory complexes and ATP synthesis were up-regulated, while genes related to the response to interferon-gamma and defense against pathogens were downregulated in atrial CMs in HF patients resulting in an increased sensitivity to SARS-CoV-2 virus infection in these atrial CMs (Supplementary Figure 1A).

**Figure 2:**
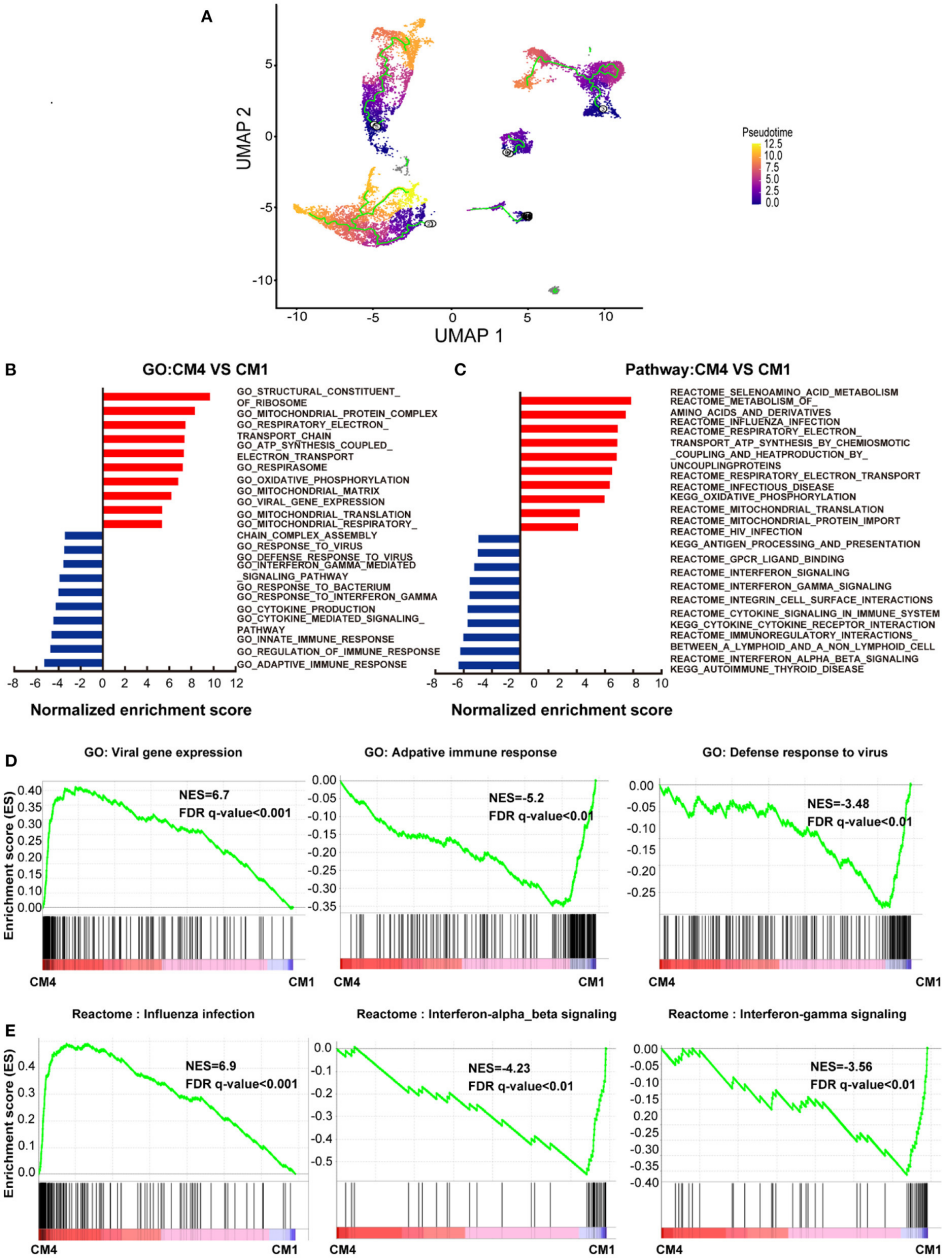
Cardiomyocytes 4 (CM4) shows different characteristics with Cardiomyocytes 1(CM1). **(A)** Pseudotime analysis of the nine clusters, the color from purple to yellow denote the different developing stage, and the simultaneous principal curve indicates the pseudo-time stage. **(B,C)** GSEA analysis revealed significant enrichment of GO and pathways for CM4 compared with CM1. **(D)** GO enrichment showing GO terms of increased viral gene expression, decreased adaptive immune response, and defense response to virus. **(E)** Influenza infection signaling pathway is up-regulated, both interferon-alpha-beta signaling and interferon-gamma signaling are down-regulated.

To further characterize this unusual CM4 subset observed in failing hearts, we performed trajectories analysis of the integrated clusters to show the pseudotime of CMs and NCMs indicating that CM4 originated from CM1 (Figure 2A). We then conducted GSEA analysis (GO and Pathway) on DEG between CM4 and CM1. GO term “Viral Gene Expression”, as well as pathways related to influenza infection were upregulated in CM4 (Figures 2B,C); while response to virus, response to interferon gamma and innate immune response, pathway of the adaptive immune response and interferon signaling were significantly down-regulated in CM4 (Figures 2D,E). Together, these results suggest that the CM4 subset predominantly observed in HF tissues would be more vulnerable to virus infection than the CM1 subset.

### Both CMs and NCMs Have Different ACE2 Expression Pattern After HF

**Figure 3:**
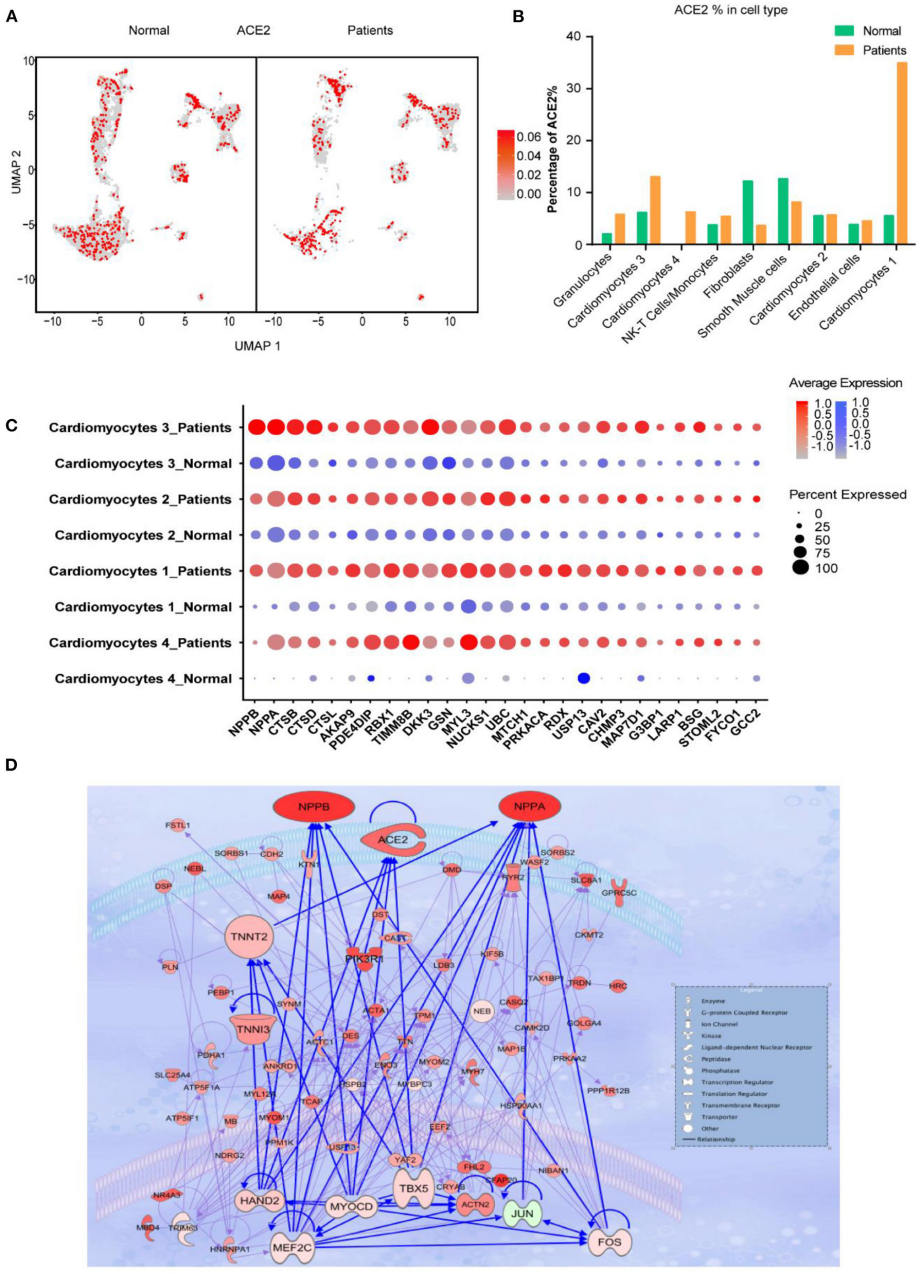
Cardiomyocytes (CMs) and Non-CMs (NCMs) have different ACE2 expression pattern. **(A)** UAMP of the CMs and NCMs subsets in normal and HF patients. **(B)** Frequency of ACE2+ cells in different cell types. **(C)** Gene expression pattern of virus infection-related genes in different subsets of CMs during HF. **(D)** Gene regulatory network of ACE2, NPPA, NPPB, and TNNT1,2,3 and their upstream binding transcription factor of HAND2, MYOCD, MEF2C, and TBX5.

We further investigated the frequency of ACE2+ cells in CMs and NCMs in normal and failing hearts. Figure 3A showed the overall distribution of ACE2+ cells in different subsets. The frequency of ACE2+ cells increased significantly in three of four CMs subsets in HF patients, especially in CM1(*p* < 0.0001) and CM4 (*p* < 0.0001), while its frequency in CM2 subset did not change significantly (*p* > 0.05; Figure 3B). Moreover, the percentages of ACE2+ cells in fibroblasts (*p* < 0.0001) and smooth muscle cells (*p* = 0.0104) were both significantly decreased. The frequency of ACE2+ cells in NK-T Cell/Monocytes and granulocytes was insignificantly changed (*p* > 0.05; Supplementary Table 2).

Taken together, scRNA-seq results demonstrated that the ACE2+ CMs dramatically increased during HF, suggesting that CMs in HF patients may be more susceptible to SARS-CoV-2 virus infection than the normal subjects. In addition, ventricular myocytes had a higher percentage of ACE2+ cells than that of atrial myocytes, indicating that these cardiomyocyte subsets may have different responses to SARS-CoV-2 infection.

### Virus Infection-Related Genes Are Upregulated in CMs in HF Patients

We then focused on gene expression dynamics of the SARS-CoV-2 entry receptor ACE2. To further examine the potential role of ACE2+ cells, we separated each cardiomyocyte subset into two sub-groups according to the expression of ACE2 (ACE2+ and ACE2-) and called DEGs between these two groups.

One of the most interesting findings was that *NPs* (*NPPA* and *NPPB)* were the top two upregulated genes in ACE2+ cells as compared to ACE2- cells. Previous studies reported that ACE2, NPs, TnT and TnI could make a feedback loop to preserve ejection fraction in HF patients (19–22). Interestingly, most of the ejection fraction preservation genes were significantly upregulated during HF, especially in ACE2+ CMs cells (Figure 3C). We used the top 100 DEGs of ACE2+ and ACE2- in CM1,4 to build a gene regulatory network (GRN) using IPA (Ingenuity Pathway Analysis, QIAGEN, CA, USA). GRN showed that *ACE2, NPs, AGT, TNNT1, TNNT2*, and *TNNT3* were well connected and shared the same upstream binding transcription factors HAND2, MYOCD, MEF2C, TBX5 which are the well-known transcription factors that can control the reprogramming of fibroblasts into CMs (Figure 3D) (23, 24). The above findings suggest that SARS-CoV-2 infection could damage CM, which in turn leads to feedback upregulation of cardio- differentiation.

**Figure 4:**
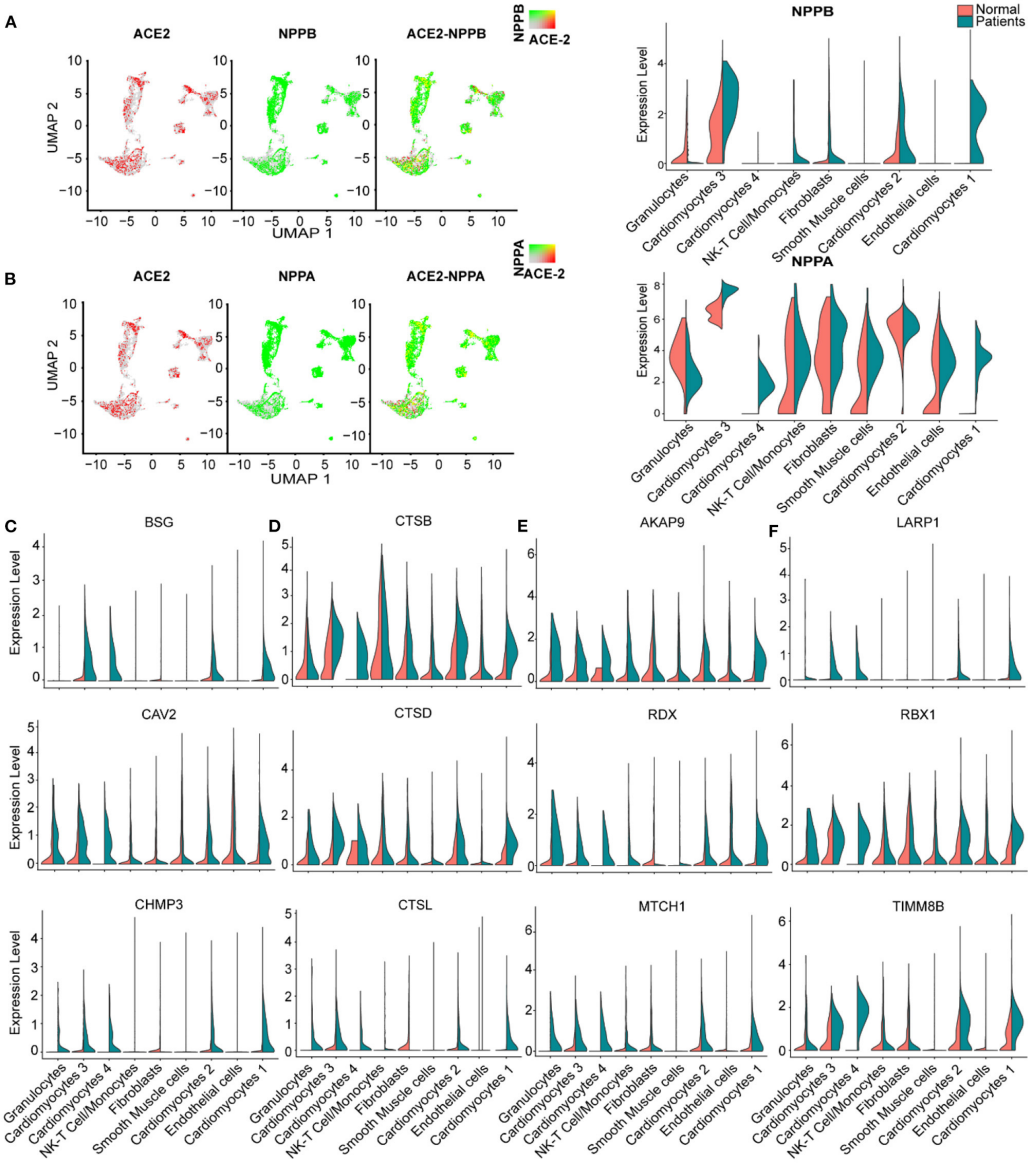
Virus related genes are upregulated in heart failure (HF) patients compared with normal. **(A)**, Expression level of ACE2 (red dots), NPPB (green dots) in different clusters, overlapping is shown in the right panel, and the co-expression is shown in yellow dots. Violin plots of the distribution of NPPB between normal and HF patients in different subsets. **(B)** Expression level of ACE2 (red dot), NPPA (green dot) in different subsets, overlapping is shown in the right panel, and the co-expression is shown in yellow dots. Violin plots of the distribution of NPPA between normal and HF patients in different subsets. **(C)** Violin plots of the distribution of genes (from top to bottom BSG, CAV2, CHMP3) related to viral infection. **(D)** Violin plots of the gene expression pattern of CST B/L. **(E)** Violin plots of the distribution of genes (from top to bottom AKAP9, RDX, MTCH1) related to IFN-γ signaling pathway. **(F)** Violin plots of the distribution of genes (from top to bottom LARP1, RBX1, and TIMM8B) on viral replication.

We further studied the expression dynamics of *ACE2* and *NPs* in CMs and NCMs in normal and HF patients. Both *NPPB* and *NPPA* were co-expressed with *ACE2* and significantly up-regulated in CMs in HF samples (Figures 4A,B), but *NPPB* and *NPPA* showed different expression patterns. Specifically, *NPPA* was expressed only in CM2, 3 and NCMs in normal heart. *NPPA* was expressed in all CMs and NCMs and its expression was significantly upregulated in all cardiomyocyte subsets after HF (Figure 4B). *NPPB* was only expressed in CM2 and CM3 subsets in normal heart, and its expression was significantly upregulated in CMs except CM4 after HF (Figure 4A). Pro-ANP can be processed by corin and pro-BNP by corin and intracellular endoprotease furin in *in vitro* experiments to form active ANP and BNP (25, 26). We found that in HF patients, corin expression increased significantly in CMs while the change for furin was insignificant (Supplementary Figure 2A), which is consistent with the observation that furin activity, but not its concentration, increased (27). Importantly, at the S1/S2 boundary of SARS-CoV-2, a furin cleavage site has been identified, which can enhance the binding of spike protein and host cells (28). It was reported that Polypeptide N-Acetylgalactosaminyl transferase, such as B3GALNT1, GALNT1 can mediate the glycosylation of pro-BNP and increase pro-BNP secretion in human cardiac during HF (29). Both B3GALNT1 and GALNT1 transcription increased in HF patients (Supplementary Figure 2B). We then assessed other virus infection-related genes, and found that genes contributing to virus entry (BSG, CAV2, CHMP3, CHMP5, and STOML2, Figure 4C, Supplementary Figures 2C,D), cysteine proteases cathepsins (CSTB, CSTD, and CSTL, Figure 4D), suppression of IFN-γ signaling (LARP1, RBX1, and TIMM8B, Figure 4E), and virus replication (AKAP9, RDX, and MTCH1, Figure 4F) were all up-regulated in CMs in failing hearts.

It was reported that SARS-CoV-2 enters host cells through the binding of its spike protein with ACE2 and subsequent S protein priming by host cell protease TMPRSS2 (30, 31).We barely detected any expression of TMPRSS2 in both normal and HF samples (Supplementary Figure 2E). Since it is reported that in the absence of cell surface protease TMPRSS2, SARS-CoV can achieve cell entry via an endosomal pathway in which it can be activated by other proteases such as cathepsin L (31), we further investigated gene expression dynamics of the endosomal cysteine proteases, cathepsins and found out that CTSB, CTSD, and CTSL were up-regulated significantly in CMs during HF (Figure 4D). Also, some inflammatory cytokines were detected and found increased in several subsets in the HF patients, such as CXCL8 which was significantly increased in the subset of granulocytes and NK-T cell/Monocytes as well as IL-32 which was increased in the subsets of NK-T cell/Monocytes and endothelial cells, respectively (Supplementary Figure 2F). Thus, we speculate that SARS-CoV-2 may use the ACE2-CTSB/L axis for cell entry in cardiac tissues. Together, these findings suggest that failing hearts might be more vulnerable to SARS-CoV-2 infection.

Thrombosis is commonly observed in severe COVID-19 patients (32). Tissue factor (TF/CD142) activation causes thrombus formation on atherosclerotic plaques coded by F3 (33). We found that blood clotting- related gene F3 was co-expressed with ACE2 and significantly up-regulated in CM3 and CM1 during HF (Supplementary Figures 2G,H), suggesting that increased F3 and ACE2 may contribute to the increased risk of thrombosis in HF patients.

### Characteristics of ACE2-Positive Ventricular and Atrial CMs, and NCMs

**Figure 5:**
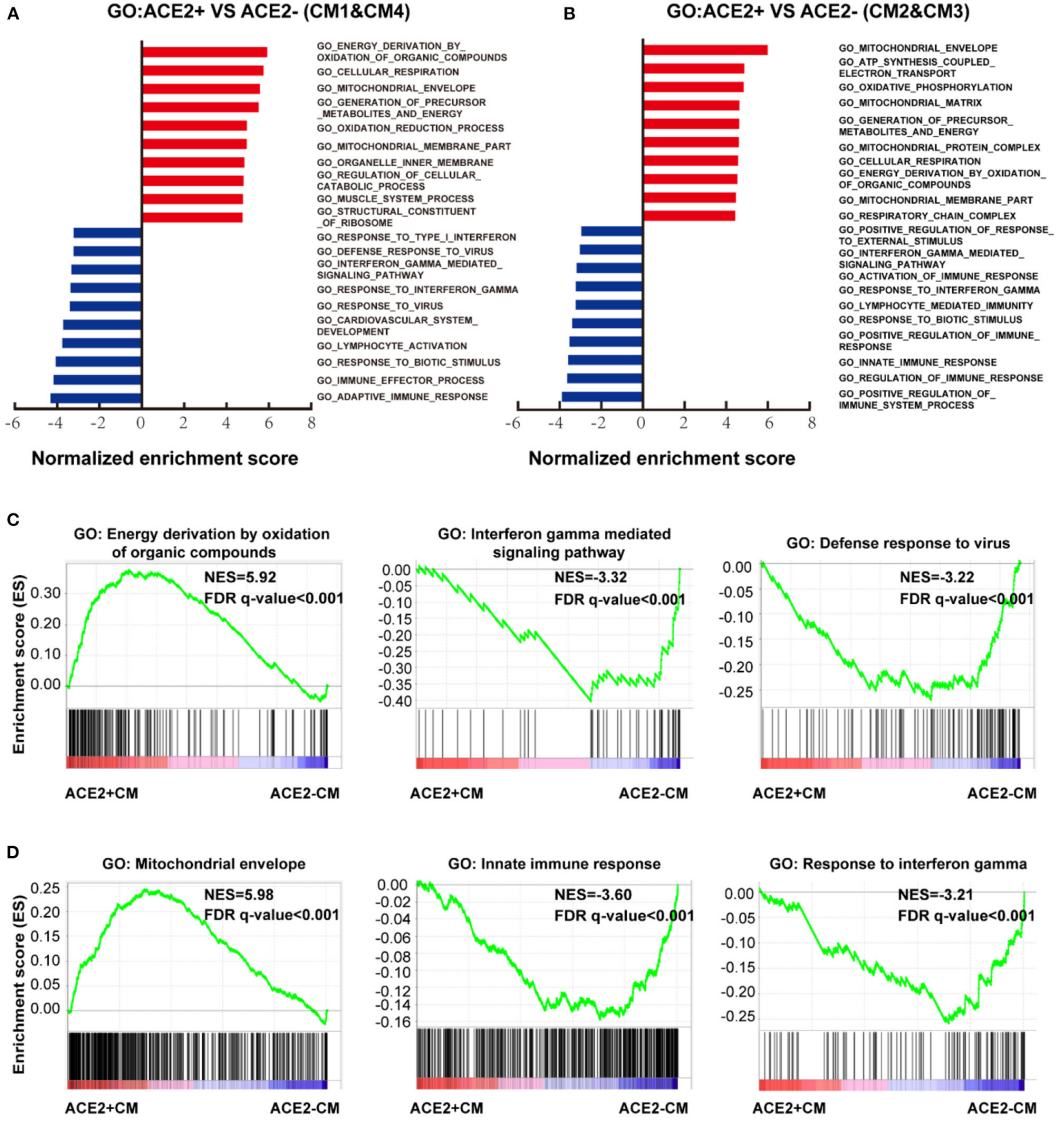
Characteristics of ACE2+ ventricular and atrial myocytes. **(A)** GO analysis revealed significant enrichment of biological pathways for ACE2+ compared with ACE2- in ventricular myocytes. **(B)** GO analysis revealed significant enrichment of biological pathways for ACE2+ compared with ACE2- in atrial myocytes. **(C)** GO plots showing GO terms of increased energy derivation by oxidation of organic compounds (left), decreased interferon gamma mediated signaling pathway (median), and down-regulated defense response to virus (right). **(D)** GO enrichment plots showing GO terms of increased mitochondrial envelope (left), decreased innate immune response (median), and down-regulated innate immune response (right). The NES and false discovery rate (FDR) were showed in panel.

We further conducted GSEA analysis on DEGs of cells between ACE2+ and ACE2- in all CMs (Figures 5A,B, Supplementary Figures 3A,B). GO terms associated with energy consumption (Figure 5A), energy derivation by oxidation (Figure 5C), and pathway influenza infection (Supplementary Figure 3C) were positively enriched in ACE2+ CM1&4; GO terms associated with energy consumption, mitochondrial envelope (Figure 5D), and pathway respiratory electron transport (Supplementary Figure 3D) were positively enriched in CM2&3. In contrast, GO terms associated with interferon gamma-mediated signaling pathway, defense response to virus, innate immune response and pathway interferon signaling were negatively enriched in ACE2+ CMs (Figures 5C,D, Supplementary Figures 3C,D).

Moreover, we also identified DEGs between ACE2+ NCMs and ACE2- NCMs and performed GSEA analysis on them (Supplementary Figure 4). Interestingly, pathways associated with infectious disease were positively enriched in NCMs, except for NK-T Cells/Monocytes. GO terms associated with mitochondrial matrix and ATP synthesis were positively enriched in smooth muscle cells, NK-T Cells/Monocytes and fibroblasts, which is consistent with the observation at CMs. GO term associated with muscle structure and function (Supplementary Figures 4A,E) and leukocyte mediated immunity were negatively enriched in ACE2+ cells of smooth muscle cells, fibroblasts, and endothelial cells (Supplementary Figure 4B). GO term associated with viral expression is positively enriched in ACE2+ granulocytes, while GO term associated with immunocyte mediated immunity is negatively enriched in ACE2+ granulocytes and ACE2+ NK-T Cells/Monocytes. These findings suggest an impaired cellular immunological response in HF patients, which may increase their vulnerability to various pathogens (Supplementary Figures 4C,D).

### Clinical Characteristics of COVID-19 Patients

**Figure 6:**
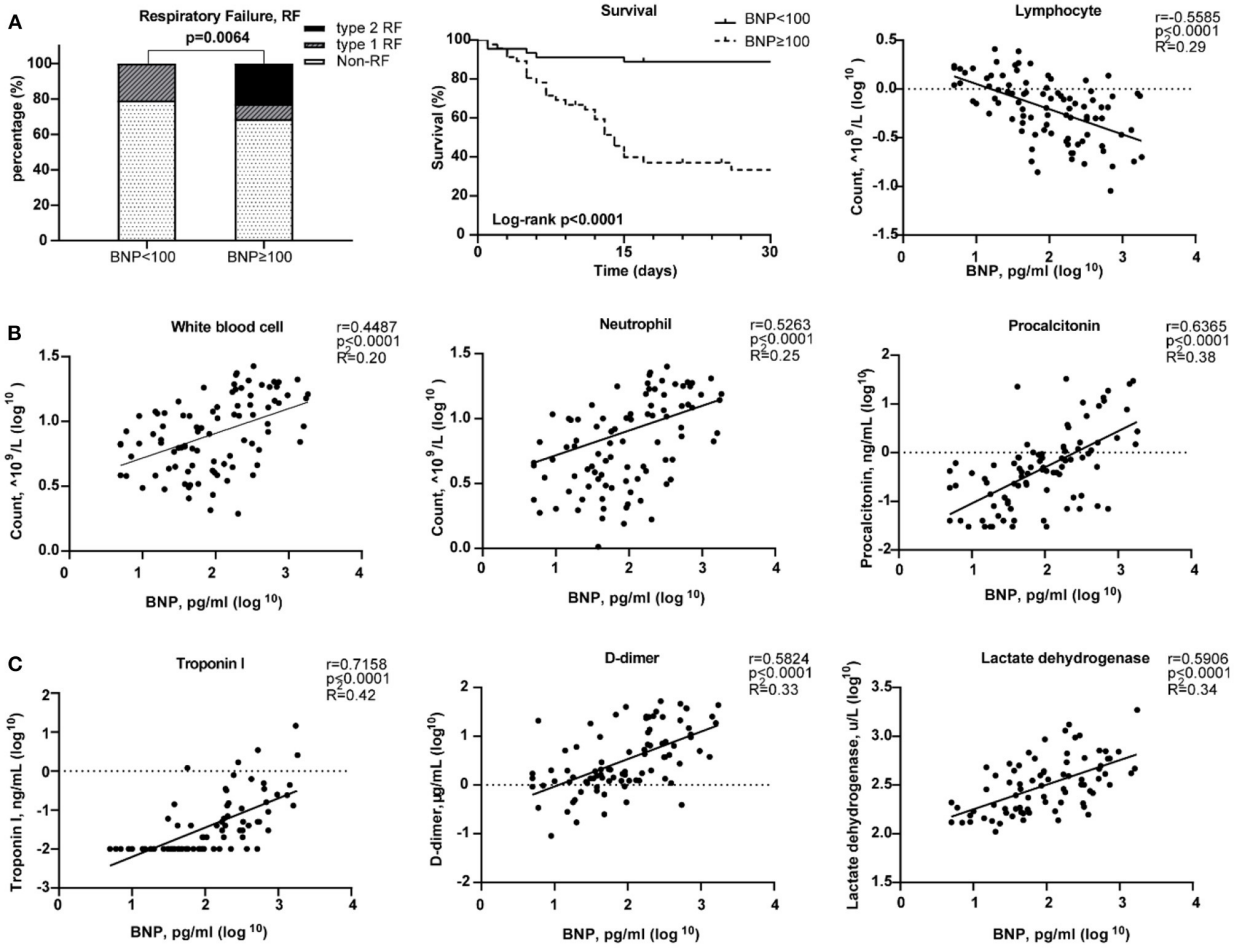
Relationships between Brain natriuretic peptide (BNP) level and clinical assessments. **(A)** Measurement of disease severity. Left figure described the constitution of non-respiratory failure (RF), type 1 RF, and type 2 RF patients. Middle figure showed K–M estimation of the mortality in high BNP group. Right figure showed the significant negative correlation of lymphocyte count and BNP level. **(B)** The severity of infection. From left to right, white blood cell, neutrophil, and lymphocyte counts were positively correlated with BNP level. **(C)** The relationship between organ impairment and BNP. Left figure depicted the strong positive relationship of cardiac injury and blood BNP level. Middle figure showed the disturbance of coagulation as BNP level increased. Right figure showed the positive correlation between lactate dehydrogenase and BNP level.

The median age of these 91 COVID-19 patients was 66 years [range, (27–89)]. Forty-six patients (50.5%) have elevated BNP (≥100 pg/mL). Both BNP level [56.0 (29.5, 259.0) vs. 105.5 (34.75, 307.3), *p* = 0.309) and proportion of higher BNP (57.41 vs. 40.54%, *p* = 0.138) were similar between male and female. HF patients have increased BNP plasma concentrations which are generally co-related with the degree of cardiac dysfunction. Thus, BNP is often used as a biochemical marker for HF (34). Patients with a higher BNP were older [median age, 71 (IQR 44–89) vs. 62 (27–79), *p* < 0.0001; Table 1]. Compared with the lower BNP group, patients in the higher BNP group have significantly higher levels of white blood cells (*p* < 0.0001) and neutrophils (*p* < 0.0001), although significantly lower number of lymphocytes (*p* < 0.0001; Table 1). The high BNP group has significant increased procalcitonin (*p* < 0.0001) and C-reactive protein (*p* < 0.0001) as compared with the low BNP group (Table 1). The high BNP group also showed imbalanced electrolyte levels and aberrant coagulation profiles as compared with the low BNP group. Furthermore, more severe organ dysfunction was observed in the high BNP group, including worse liver function indicated by higher aspartate transaminase (*p* < 0.03), direct bilirubin (*p* < 0.005), and lactate dehydrogenase (*p* < 0.0001; Table 1). The high BNP group also showed worse renal function as indicated by a reduced glomerular filtration rate (*p* < 0.0003) and increased blood urea nitrogen (*p* < 0.0001; Table 1). Cardiac TNI (*p* < 0.0001) was significantly increased in the higher BNP group, suggesting more cardiac injury in these patients (Table 1). Noteworthy, the high BNP group had a higher incidence of respiratory failure (RF, 31.43%, *p* = 0.0064; Figure 6A, left), and a significantly increased mortality rate (58.70%, *p* < 0.0001; Figure 6A middle, Table 1), and a negative correlation with the lymphocyte count (Figure 6A, right). Infective markers were positively correlated with the BNP level (Figure 6B). Markers of coagulative disturbance and organ impairment were positively correlated with the BNP level (Figure 6C, middle and right).

**Table 1:** Comparison of COVID-19 patient characteristics between BNP groups.

**Parameters**	**Total (*N* = 91)**	**BNP<100 (*N* = 45)**	**BNP ≥ 100 (*N* = 46)**	***p*-value**
Age, yrs, median [min, max]	66 (27–89)	62 (27–79)	71 (44–89)	<0.0001*
Male, *n* (%)	54 (59.3)	23 (51.1)	31 (67.4)	0.11
**Complete blood cell count, 10^9^/*L***				
White blood cell, median (IQR)	7.99 (4.59–13.31)	6.28 (4.04–8.38)	13.05 (6.76–18.13)	<0.0001*
Neutrophil, median (IQR)	6.6 (3.43–12.32)	4.29 (2.74–6.67)	11.88 (4.83–16.93)	<0.0001*
Lymphocyte, median (IQR)	0.71 (0.38–1.09)	0.98 (0.62–1.47)	0.50 (0.27–0.78)	<0.0001*
**Liver and renal function**				
Alanine transaminase, U/L, median (IQR)	30.0 (18.5–52.5)	27.0 (19.0–49.0)	32.0 (18.0–64.0)	0.5783
Aspartate transaminase, U/L, median (IQR)	37.0 (23.0–55.0)	30.0 (20.0–51.0)	41.5 (29.0–64.0)	0.0299*
TBIL, μmol/L, median (IQR)	14.1 (9.5–21.7)	11.8 (9.1–17.8)	15.2 (10.1–24.4)	0.1216
Direct bilirubin, μmol/L, median (IQR)	5.3 (3.4–9.8)	4.1 (3.0–6.5)	6.6 (4.0–13.2)	0.0047*
Lactate dehydrogenase, U/L, median (IQR)	315.0 (179.5–470.5)	185.0 (154.0–352.0)	407.0 (288.0–599.0)	<0.0001*
eGFR, mL/(min*1.73 m^2^), mean±SD	105.6 ± 47.0	121.1 ± 41.6	86.5 ± 44.5	0.0003*
Blood urea nitrogen, mmol/L, median (IQR)	5.7 (3.9–11.1)	4.5 (3.2–5.8)	9.0 (5.2–15.9)	<0.0001*
Uric acid, μmol/L, median (IQR)	234.0 (183.5–305.5)	235.0 (184.0–305.0)	230.5 (182.0–310.0)	0.9494
**Cardiac biomarker**				
Troponin-I, ng/mL, median (IQR)	0.01 (0.01–0.06)	0.01 (0.01–0.01)	0.05 (0.03–0.25)	<0.0001*
**Electrolytes**				
Potassium, mmol/L, median(IQR)	4.04 (3.64–4.40)	3.87 (3.56–4.27)	4.19 (3.64–4.70)	0.0354*
Sodium, mmol/L, median (IQR)	139.0 (136.0–142.0)	139.0 (135.0–141.0)	139.0 (136.0–145.0)	0.2992
Chloride, mmol/L, median (IQR)	102.0 (98.5–106.0)	103.0 (100.0–106.0)	101.5 (98.0–106.0)	0.6473
Calcium, mmol/L, mean ± SD	2.03 ± 0.18	2.09 ± 0.16	1.97 ± 0.18	0.0018*
**Coagulation profiles**				
Prothrombin time, s, median (IQR)	13.4 (12.4–14.9)	13.0 (12.0–13.8)	13.9 (12.8–16.7)	0.0030*
APTT, s, median (IQR)	35.5 (31.8–39.8)	35.1 (32.4–38.9)	35.5 (30.7–42.5)	0.6165
Fibrinogen, g/L, median (IQR)	3.39 (2.31–4.77)	3.39 (2.31–5.09)	3.40 (2.35–5.87)	0.8567
D-dimer, μg/mL, median (IQR)	2.03 (1.22–1.00)	1.37 (0.83–1.99)	6.96 (3.25–24.20)	<0.0001*
**Inflammatory biomarkers**				
Procalcitonin, ng/mL, median(IQR)	0.45 (0.12–1.12)	0.23 (0.04–0.49)	1.01 (0.39–3.51)	<0.0001*
hsCRP, mg/L, median (IQR)	13.80 (5.74–20.50)	6.09 (1.52–15.86)	18.00 (13.45–21.50)	<0.0001*
**Blood gas analysis**				
PaO_2_, mmHg, median (IQR)	71.0 (57.8–92.0)	78.5 (57.5–104.5)	68.5 (56.5–86.0)	0.4867
PaCO_2_, mmHg, median (IQR)	41.0 (34.0–48.8)	39.5 (33.5–43.5)	42.5 (34.0–57.9)	0.1589
Lactic acid, mmol/L, median (IQR)	1.95 (1.40–2.40)	1.80(1.30–2.15)	2.00 (1.60–2.75)	0.1634
BNP, pg/mL, median (IQR)	92.0 (32.5–299.5)	34.0 (15.0–48.0)	299.5 (180.0–548.0)	<0.0001*
Death, *n* (%)	32 (35.16)	5 (11.11)	27 (58.70)	<0.0001*

*Continuous variables are presented as means ± SD if they conform to normal distribution, or median with interquartile range if not. Age is presented as median with range (minimum–maximum). Categorical variables are presented as percentage (%)*.

* *p < 0.05. TBIL, total bilirubin; eGFR, estimated glomerular filtration rate (calculated by MDRD formula); APTT, activated partial thromboplastin time; hsCRP, high-sensitive C-reactive protein; BNP, brain natriuretic peptide. Bold values means p < 0.05 which have statistics significance*.

## Discussion

**Figure 7:**
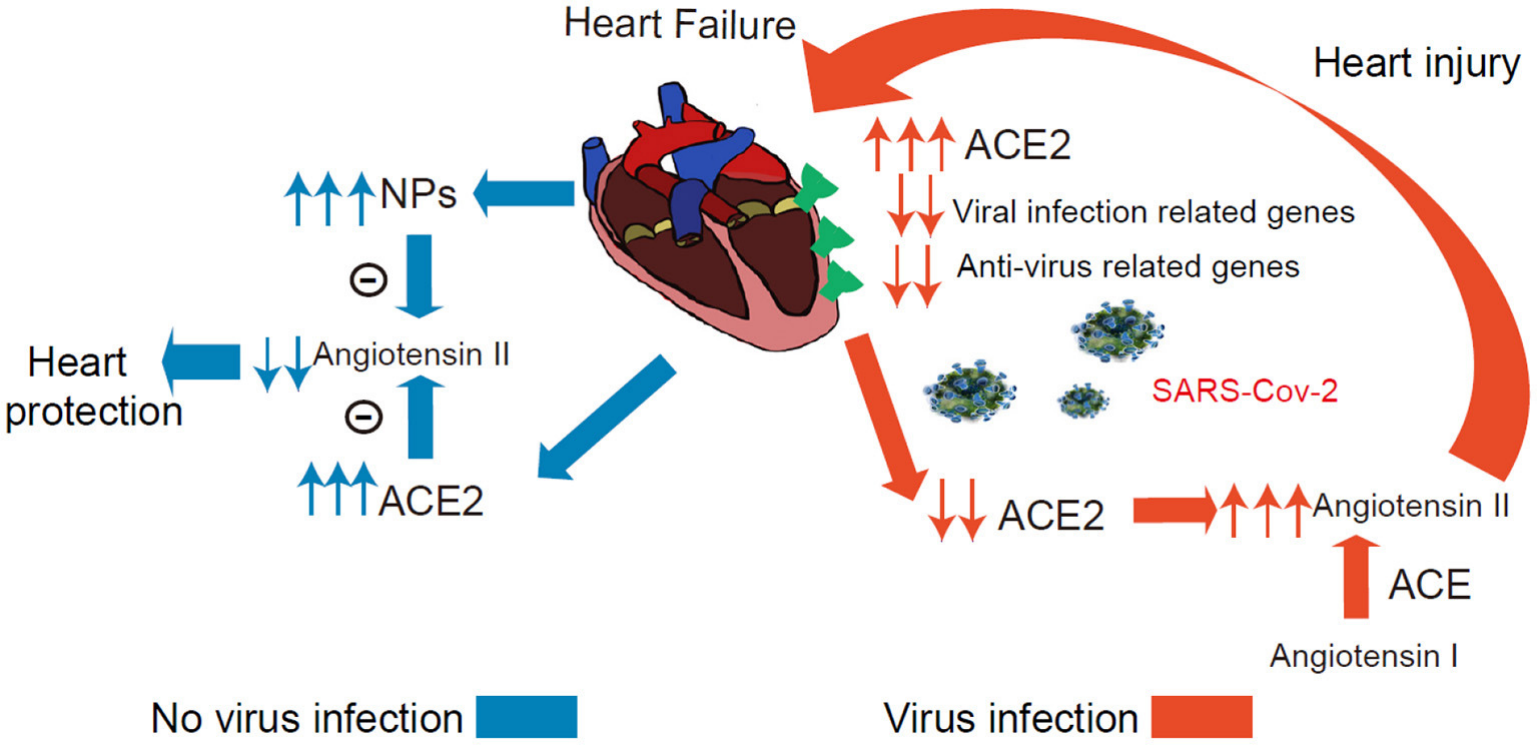
Conceptual Schematic diagram highlighting the central role of SARS-CoV-2 and the NPs, RAAS in the potentially deleterious (red) and protective (blue) effects. **Left:** Schematic diagram showed that the ACE2 and NPs can protect heart from heart failure noted in blue. **Right:** The process under virus infection was noted in red to speculate the underlying relationship for the higher susceptibility and worse prognosis in HF.

The present study has several major findings. First, the study systematically investigated the ACE2 expression dynamics in CMs, and Non-CMs in human normal and failing hearts at the single-cell level. We found that ACE2 was expressed in some CMs, vascular endothelial cells, and smooth muscle cells in both normal and failing hearts. Second, we demonstrated that ACE2 expression was selectively increased in the CM4 subset from 0 to 7.01% after heart failing, suggests that CM4 may be more vulnerable to SARS-CoV-2 infection than CM1. Third, we demonstrated for the first time that NPs transcripts are markedly enriched in ACE2+ CMs, which suggest that ACE2 and NPs may share similar signaling pathway and ACE2+NPPB+/ACE2+NPPA+ CMs may play important role in viral infection of HF patients. Fourth, we demonstrated that ACE2 expression was associated with the dynamic changes of a group of genes specific for the networks of viral infection and immunity in CMs (Figure 7).

We found that ACE2 was expressed in ~5% normal ventricular or atrial CMs (2% in lung AT2 cells) and ACE2+ cells frequency was increased in the CM1, CM3, and CM4 subset in failing hearts. Our finding that ACE2 was expressed in normal hearts appears to contradict a previous report that pericytes, but not the CMs express ACE2 in normal hearts (35). The discrepancy may due to the fact that the previous study used the single nucleus RNA-seq approach, which generally captures fewer transcripts as compared with the more sensitive and comprehensive SMART-seq using whole-cell in our study. In the context that SARS-CoV-2 causes myocarditis and cardiac injury (36), it is reasonable to believe that the increased ACE2+ in CMs in the failing heart could make these CMs vulnerable to SARS-CoV-2 infection in COVID-19 patients.

Several studies have defined a critical role for ACE2 in protecting the heart against HF, systemic and pulmonary hypertension, myocardial infarction, and diabetic cardiomyopathy (18, 36, 37). Another very interesting finding is that both NPs transcripts were markedly enriched in ACE2+ CMs, and that NPs and ACE2 can form two negative feedback loops respectively associated with the RAAS/Ang II signaling pathway (38). Since ACE2 degrade Ang-II, the expression of ACE2 in CMs is likely to protect these CMs through reducing local Ang-II content under conditions without SARS-CoV-2 infection. Circulating NPs can promote diuresis, natriuresis and vasodilation, which is critical for the maintenance of intravascular volume homeostasis (Figure 7) (19). In addition, GRN showed that ACE2 and NPs might be co-regulated during HF development.

SARS-CoV-2 binding to ACE2 could result in ACE2 degradation or dysfunction (37, 38). A recent study demonstrated that the plasma Ang-II level from SARS-CoV-2 infected patients was markedly elevated and the plasma Ang-II linearly correlated with the viral load and lung injury in COVID-19 patients (2, 39, 40). Our clinical data indicate that COVID-19 patients with a higher level of BNP had a more severe dysfunction of the heart and significantly higher mortality. Whether this increased BNP leads to higher mortality or constitutes an endogenous cardioprotective strategy in the settings of SARS-CoV-2-mediated inflammation remains to be confirmed. As patients are infected by SARS-CoV-2 in cardiac tissues, the overall virus defense capacity would be attenuated in CMs (Figure 7), SARS-CoV-2 infection in ACE2+ CMs could certainly cause or exacerbate cardiac injury and consequent cardiac dysfunction. Drug ACEI or ARBs can break this positive feedback loop by reducing Ang II (Figure 7). Importantly, another study found that plasma ACE2 was not related to ACEI or ARBs use in HF patients which alludes to the fact that using ACEI or ARBs will not increase the risk of virus infection (41). We speculate that patients may benefit from these types of drugs partly attributed to these explanations. Indeed, another study demonstrated that in hospitalized COVID-19 patients with hypertension, patient's use of ACEI/ARB was associated with lower risk of all-cause mortality compared with ACEI/ARB non-users (38, 42, 43).

In the failing heart, the upregulation of ACE2, virus infection and oxidative phosphorylation associated genes could facilitate SARS-CoV-2 virus entry and replication. These findings may advance our understanding of the underlying pathobiology of myocarditis associated with COVID-19 and new treatment strategy. The direct relationship between ACE2 and NPs and the role of ACE2+NPPB+/ACE2+NPPA+ CMs in viral infection would be the focus of our future studies. We will also further explore the new marker of failing CMs, especially CM4, and the mechanism of the virus susceptibility in failing hearts.

## Data Availability Statement

The original contributions presented in the study are included in the article/Supplementary Material, further inquiries can be directed to the corresponding author/s.

## Ethics Statement

The studies involving human participants were reviewed and approved by Approval No. L2020-Y-013 and No. SHSY-IEC-4.1/20-63/02. The patients/participants provided their written informed consent to participate in this study. Written informed consent was obtained from the individual(s) for the publication of any potentially identifiable images or data included in this article.

## Author Contributions

XX and DX: conception and design. XX: scRNA-seq data collection and analysis. XH, MC, and MZ: provision of study materials or patients, collection, and assembly of clinical data. XX, MM, YX, DX, YS, YC, S-BO, and HL: results interpretation and manuscript writing. XX, DX, YC, HL, S-BO, YS, MM, YX, XH, MC, and MZ: final approval of manuscript. All authors contributed to the article and approved the submitted version.

## Conflict of Interest

The authors declare that the research was conducted in the absence of any commercial or financial relationships that could be construed as a potential conflict of interest.
