# Supplementary figures and images for: Single-Cell Transcriptome Analysis Decipher New Potential Regulation Mechanism of ACE2 and NPs Signaling Among Heart Failure Patients Infected With SARS-CoV-2
Source: Front Cardiovasc Med. 2021 Feb 23;8:628885. doi: 10.3389/fcvm.2021.628885 (PMC7952310; doi:10.3389/fcvm.2021.628885)

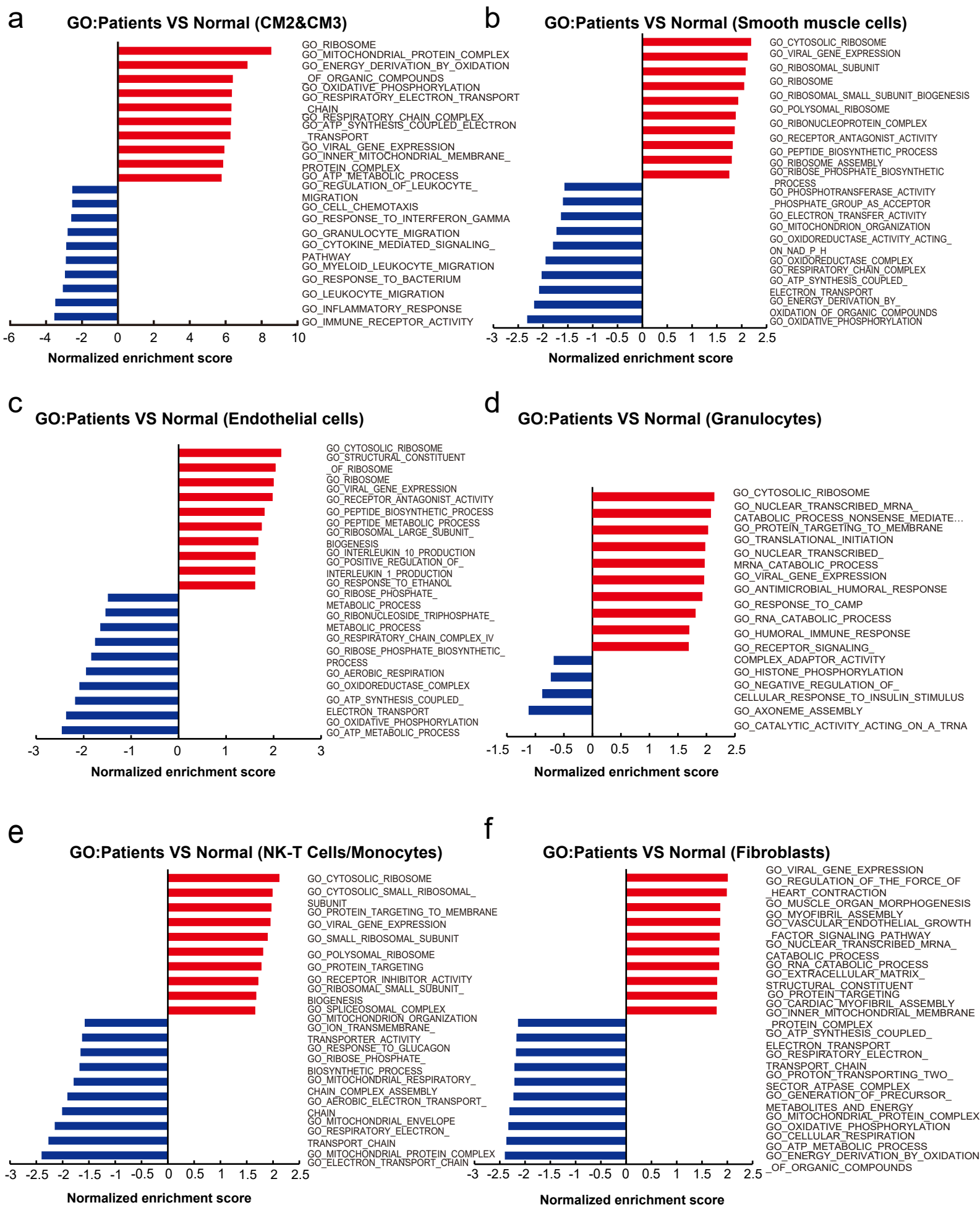

Supplement: Supplementary file 1 [file Image_1.pdf]

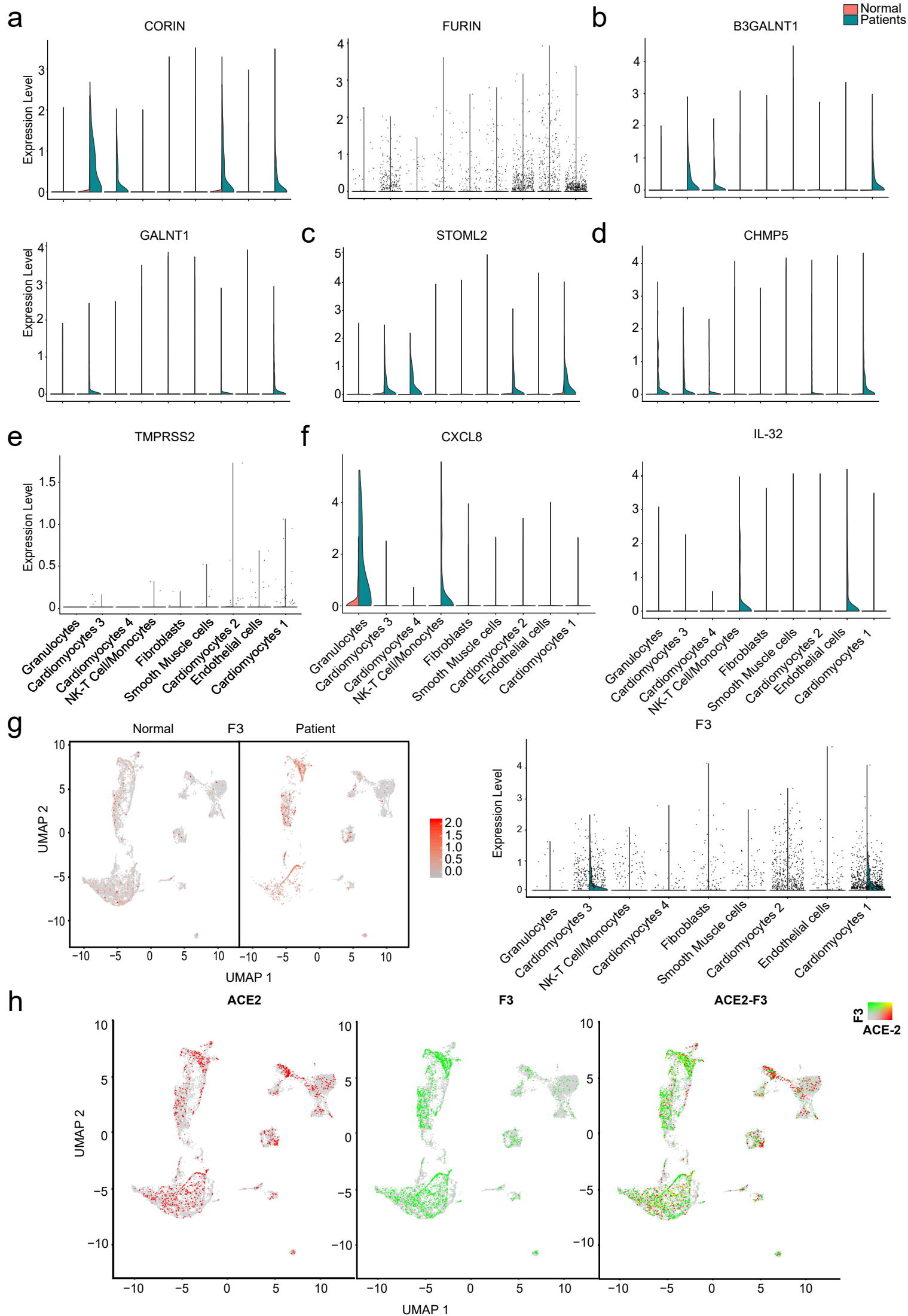

Supplement: Supplementary file 2 [file Image_2.pdf]

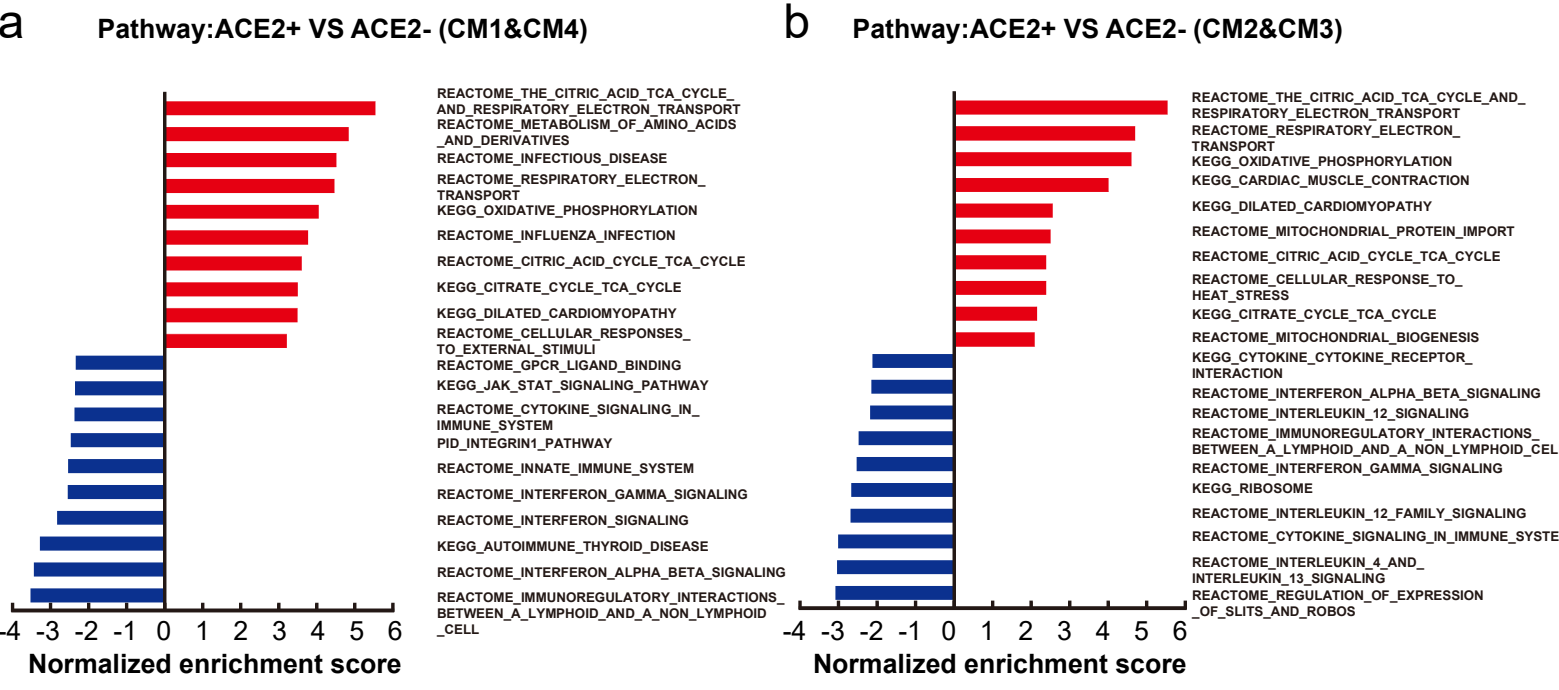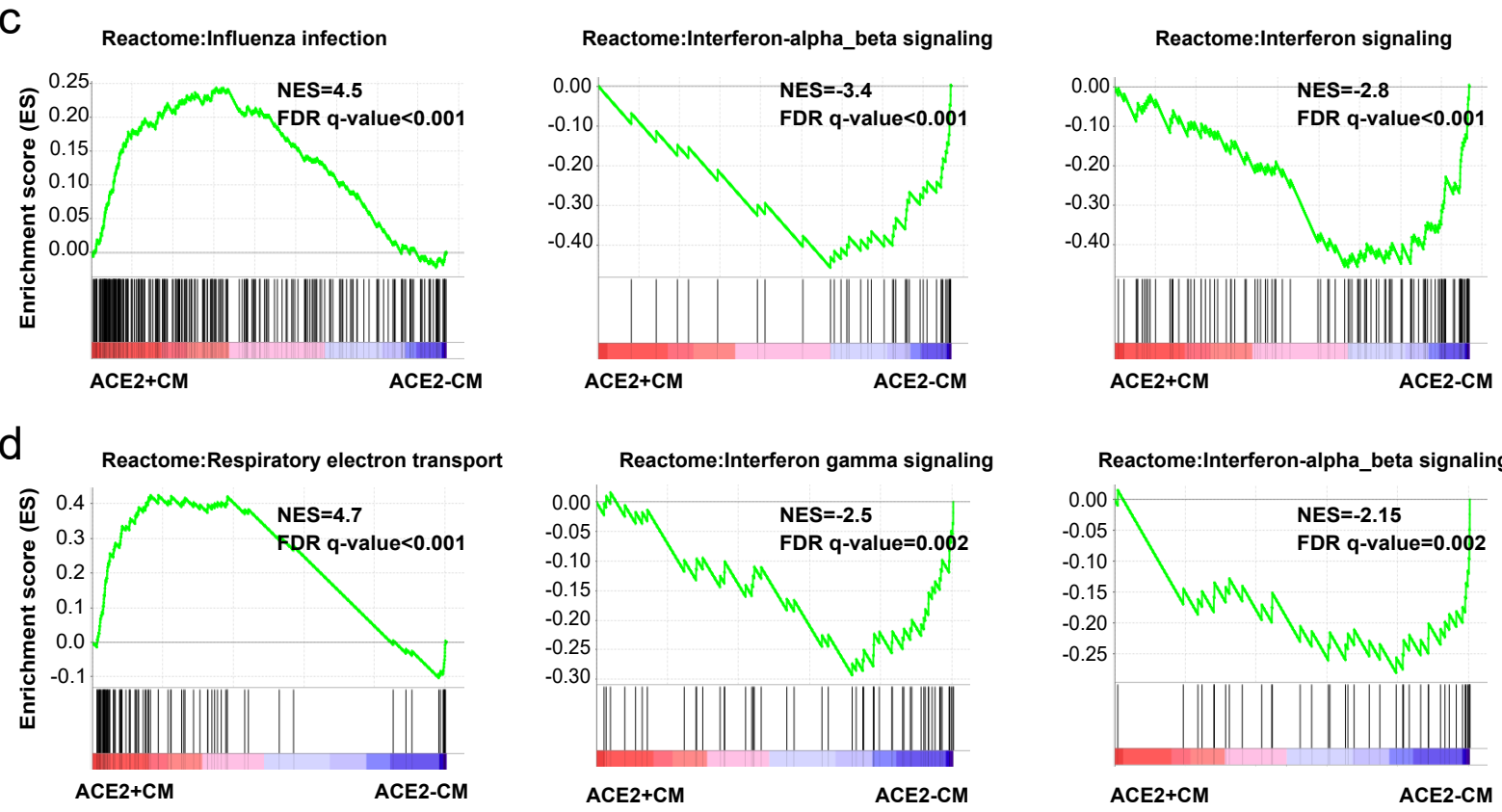

Supplement: Supplementary file 3 [file Image_3.pdf]

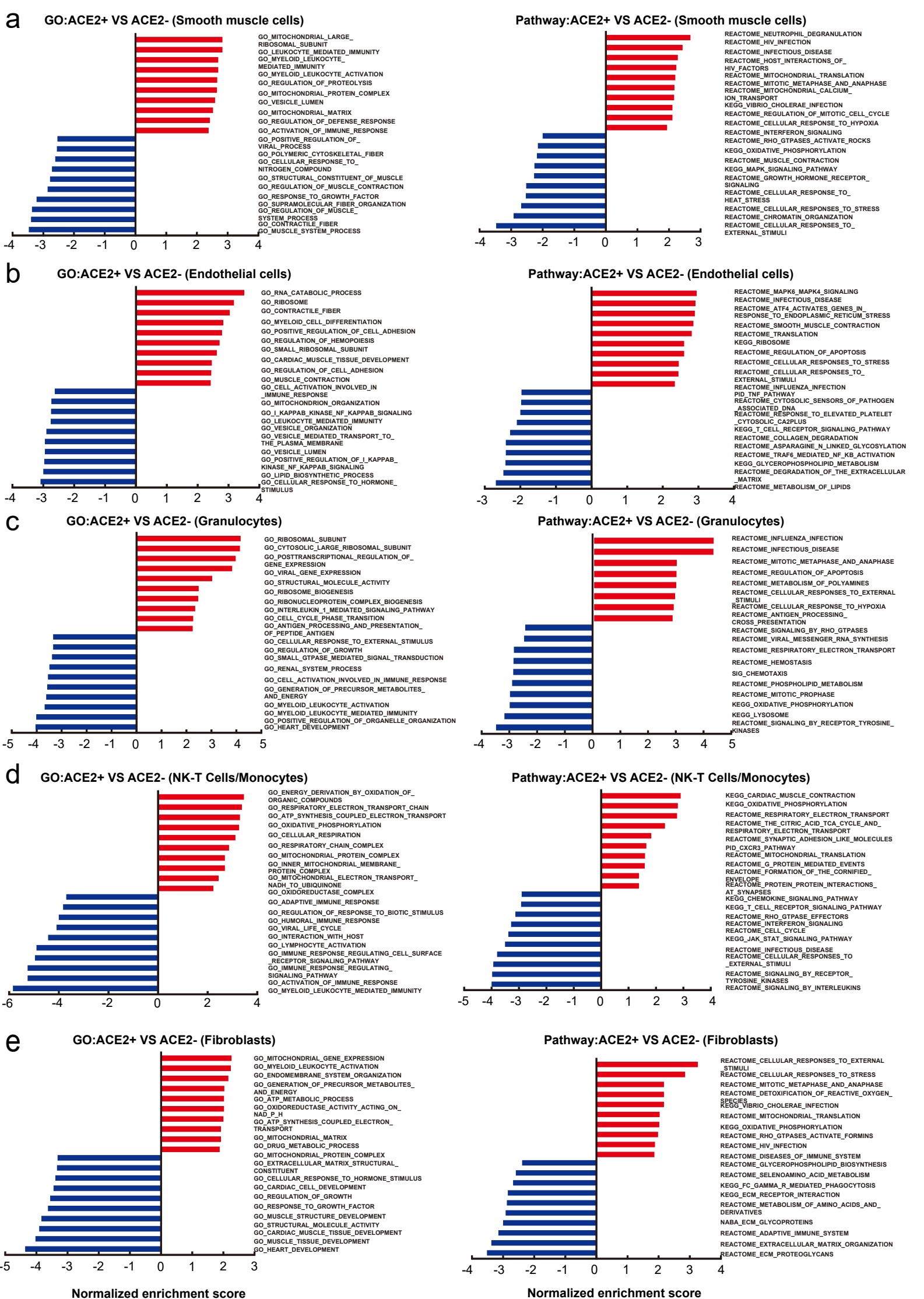

Supplement: Supplementary file 4 [file Image_4.pdf]
