# Supplementary material for: Single-Cell Transcriptome Analysis Decipher New Potential Regulation Mechanism of ACE2 and NPs Signaling Among Heart Failure Patients Infected With SARS-CoV-2
Source: Front Cardiovasc Med. 2021 Feb 23;8:628885. doi: 10.3389/fcvm.2021.628885 (PMC7952310; doi:10.3389/fcvm.2021.628885)
Supplement: Supplementary file 5 [file Table_1.docx]

| Cell type | Normal % | Patients % | P-Value |
| --- | --- | --- | --- |
| Granulocytes | 1.51 | 2.44 | p> 0.05 |
| Cardiomyocytes 3 | 8.27 | 8.53 | p>0.05 |
| Cardiomyocytes 4 | 0.03 | 8.70 | p<0.0001 |
| NK-T Cell/Monocytes | 3.53 | 4.81 | p> 0.05 |
| Fibroblasts | 4.53 | 8.41 | p<0.0001 |
| Smooth Muscle | 7.99 | 13.58 | p<0.0001 |
| Cardiomyocytes 2 | 17.70 | 18.68 | p>0.05 |
| Endothelial | 16.79 | 28.13 | p<0.0001 |
| Cardiomyocytes 1 | 39.65 | 6.71 | p<0.0001 |

Supplementary Table 1 The differential distribution of subsets between normal and HF patients.
