# Supplementary material for: Single-Cell Transcriptome Analysis Decipher New Potential Regulation Mechanism of ACE2 and NPs Signaling Among Heart Failure Patients Infected With SARS-CoV-2
Source: Front Cardiovasc Med. 2021 Feb 23;8:628885. doi: 10.3389/fcvm.2021.628885 (PMC7952310; doi:10.3389/fcvm.2021.628885)
Supplement: Supplementary file 6 [file Table_2.docx]

| Cell type | Normal % | Patients % | P-Value |
| --- | --- | --- | --- |
| Granulocytes | 2.04 | 5.83 | p> 0.05 |
| Cardiomyocytes 3 | 6.19 | 13.06 | p<0.0001 |
| Cardiomyocytes 4 | 0.00 | 6.27 | p<0.0001 |
| NK-T Cell/Monocytes | 3.77 | 5.42 | p> 0.05 |
| Fibroblasts | 12.22 | 3.66 | p<0.0001 |
| Smooth Muscle | 12.69 | 8.20 | p=0.0104 |
| Cardiomyocytes 2 | 5.55 | 5.71 | p>0.05 |
| Endothelial | 3.84 | 4.55 | p>0.05 |
| Cardiomyocytes 1 | 5.55 | 34.98 | p<0.0001 |

Supplementary Table 2 The frequency of ACE2+ cells in CMs and NCMs of normal and HF patients.
